# Supplementary material for: The Invariance Hypothesis Implies Domain-Specific Regions in Visual Cortex
Source: PLoS Comput Biol. 2015 Oct 23;11(10):e1004390. doi: 10.1371/journal.pcbi.1004390 (PMC4619805; doi:10.1371/journal.pcbi.1004390)
Supplement: S1 Text — Text S1 (PDF) [file pcbi.1004390.s001.pdf]

Supplementary Information for: The invariance hypothesis implies domain-specific regions in visual cortex

Joel Z. Leibo*[1,2], Qianli Liao[1,2], Fabio Anselmi[1,2,3], & Tomaso Poggio[1,2,3]

[1]Center for Brains, Minds, and Machines and the McGovern Institute for Brain Research, at the Massachusetts Institute of Technology, Cambridge, MA 02139, USA
[2]McGovern Institute for Brain Research, Cambridge, MA 02139, USA
[3]Istituto Italiano di Tecnologia, Genova, 16163, Italy
*To whom correspondence should be addressed: jzleibo@mit.edu

Contents

1 Remarks on a theory of architectures for invariant recognition **2**
1.1 The first regime: generic invariance 2
1.2 The second regime: class-specific invariance 4

2 Illumination invariance **6**

3 Pose-invariant body recognition **8**

4 Development of domain-specific regions **9**

5 Supplementary methods **13**
5.1 Stimuli 13
5.2 Body-pose experiments 13
5.3 Clustering by transformation compatibility 13

1 Remarks on a theory of architectures for invariant recognition

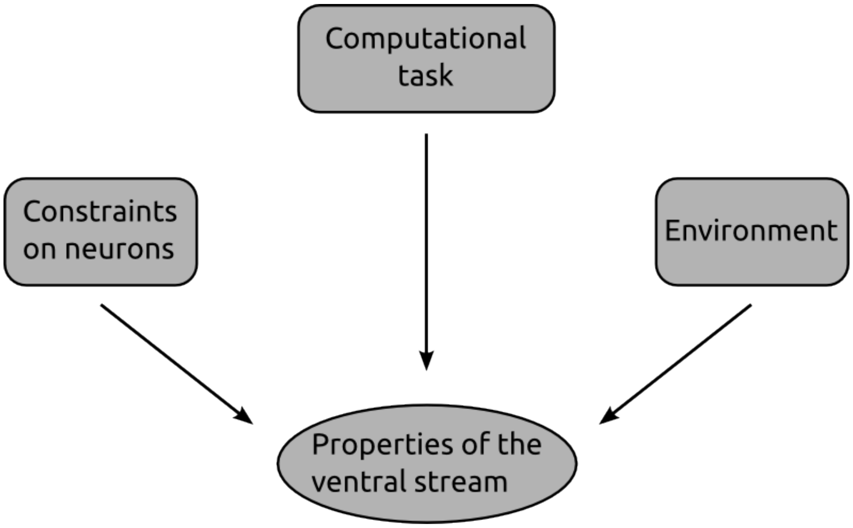

Figure 1. It is hypothesized that properties of the ventral stream are determined by these three factors. We are not the only ones to identify them in this way. For example, Simoncelli and Olshausen distinguished the same three factors [1]. The crucial difference between their *efficient coding hypothesis* and our *invariance hypothesis* is the particular computational task that we consider. In their case, the task is to provide an efficient representation of the visual world. In our case, the task is to provide an invariant signature supporting object recognition.

The new theory of architectures for object recognition [2]—applied here to the ventral stream—is quite general. It encompasses many non-biological hierarchical networks in the computer vision literature in addition to ventral stream models like HMAX. It also implies the existence of a wider class of hierarchical recognition algorithms that has not yet been fully explored. The conjecture with which this paper is concerned is that the algorithm implemented by the ventral stream's feedforward processing is in this class. The theory can be developed from four postulates: (1) Computing a representation that is unique to each object and invariant to identity-preserving transformations is the main computational problem to be solved by an object recognition system—i.e., by the ventral stream. (2) The ventral stream's feedforward, hierarchical operating mode is sufficient for recognition [3–5]. (3) Neurons can compute high-dimensional dot products between their inputs and a stored vector of synaptic weights [6]. (4) Each layer of the hierarchy implements the same basic "HW-"module, performing filtering and pooling operations via the scheme proposed by Hubel and Wiesel for the wiring of V1 simple cells to complex cells [7].

We argue that as long as these postulates are approximately correct, then the algorithm implemented by the (feedforward) ventral stream is in the class described by the theory, and this is sufficient to explain its domain-specific organization.

1.1 The first regime: generic invariance

First, consider the (compact) group of 2D in-plane rotations G. With some abuse of notation, we use g to indicate both an element of G and its unitary representation acting on images. The orbit of an image I under the action of the group is $O_I = \{gI \mid g \in G\}$. The orbit is invariant and unique to the object depicted in I. That is, $O_I = O_{I'}$ if and only if $I' = gI$ for some $g \in G$. For an example, let I be an image.

Its orbit O_I is the set of all images obtained by rotating I in plane. Now consider, $g_{90^\circ}I$, its rotation by 90°. The two orbits are clearly the same, i.e. $O_I = O_{g_{90^\circ}I}$. The set of images obtained by rotating I is the same as the set of images obtained by rotating $g_{90^\circ}I$.

The fact that orbits are invariant and unique (for compact groups) suggests a recognition strategy. Simply store the orbit for each familiar object. Then, for each new image, check what orbit it is in. Such a strategy would yield invariant representations for familiar objects. However, it could only be used in cases where we had already stored the entire orbit for all objects of interest. How could this approach work in the more realistic setting where only one sample from the test object's orbit is available?

The key property that enables this approach to object recognition is the following condition. For a stored *template* t with unit norm

$$\langle gI, t\rangle = \langle I, g't\rangle \quad \exists g' \in G \;\; \forall g \in G. \tag{1}$$

It is true whenever g is unitary since in that case $g' = g^{-1}$. It implies that it is not necessary to have the orbit of I in advance. Instead, the orbit of t is sufficient. Eq. (1) enables the invariance learned from observing a set of templates to transfer to new images. Consider the case where the full orbits of several templates $t_1, \ldots, t_K$ were stored. Let I be a completely novel image. Let P be a function mapping sets of real numbers to $\mathbb{R}$. For example, we can choose $P = \max(\cdot)$. An invariant signature $\mu(\cdot)$ can be defined as

$$\mu(I) = \begin{pmatrix} P\left(\{\langle I, gt_1\rangle \;\mid g \in G\}\right) \\ \vdots \\ P\left(\{\langle I, gt_K\rangle \;\mid g \in G\}\right) \end{pmatrix}. \tag{2}$$

So far, this analysis has only applied to compact groups. Essentially the only interesting one is in-plane rotation. We need an additional idea in order to consider more general groups: Most transformations are generally only observed through a range of transformation parameters. For example, in principle, one could translate arbitrary distances. But in practice, all translations are contained within some finite window. That is, rather than considering the full orbit under the action of G, we consider partial orbits under the action of a subset $G_0 \subset G$ (note: G_0 is not a subgroup).

We can now define the basic module that will repeat through the hierarchy. As mentioned in the main text, an HW-module consists of one C-unit and all of its afferent S-units. For an image I, the output of the k-th HW-module is $\mu_k(I) = P(\{\langle I, gt_k\rangle) \mid g \in G_0\})$. The subset G_0 is called the HW-module's pooling domain. Note that if G_0 is a set of translations the pooling domain has the same interpretation as a spatial region as in HMAX.

Consider, for simplicity, the case of 1D images (centered in zero) transforming under the 1D locally compact group of translations. What are the conditions under which an HW-module will be invariant over the range $G_0 = [-b, b]$? Let $P(\cdot) := \sum_{x\in[-b,b]} \eta(\cdot)$, where η is a positive, bijective function. The k-th component of the signature vector will then be

$$\mu_k(I) = \sum_{x\in[-b,b]} \eta\Big(\langle I, T_x t_k\rangle \Big)$$

where T_x is the operator acting on a function f as $T_x f(x') = f(x' - x)$. Suppose we transform the image I (or equivalently, the template) by a translation of $\bar{x} > 0$, implemented by $T_{\bar{x}}$. Under what conditions does $\mu_k(I) = \mu_k(T_{\bar{x}}I)$? Note first that $\langle I, T_x t_k\rangle = (I * t_k)(x)$, where $*$ indicates convolution. By the properties of the convolution operator, we have $[(T_{\bar{x}}I) * t_k](x) = T_{\bar{x}}(I * t_k)(x)$ which implies

$$\text{supp}[(T_{\bar{x}}I) * t_k] = T_{\bar{x}}\text{supp}(I * t_k).$$

This observation allows us to write a condition for the invariance of the signature vector components with respect to the translation $T_{\bar{x}}$ (see also Fig. 2). For a positive nonlinearity η, (no cancelations in the sum)

and bijective (the support of the dot product is unchanged by applying η) the condition for invariance is:

$$T_{\bar{x}}\text{supp}(\langle I, T_x t_k \rangle) \subseteq [-b, b] \tag{3}$$

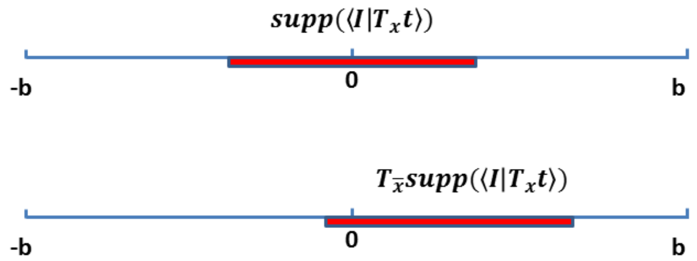

Figure 2. Localization condition of the S-unit response for invariance under the transformation $T_{\bar{x}}$

Eq. 3 is a localization condition on the S-unit response. It is necessary and sufficient for invariance. In this case, eq. (1) is trivial since we are considering group transformations.

1.2 The second regime: class-specific invariance

So far, we have explained how the localization properties of the S-response allow invariance in the case of partially observed group transformations. Next, we show how localization still enables approximate invariance (ϵ-invariance) even in the case of non-group (smooth) transformations. However, as will be shown below, in order for eq. (1) to be (approximately) satisfied, the class of templates needs to be much more constrained than in the group case.

Consider a smooth transformation parametrized by $r \in \mathbb{R}$, T_r. Its Taylor expansion w.r.t. r around, e.g., zero is:

$$T_r(I) = T_0(I) + J^I(I)r + O(r^2) = I + J^I(I)r + O(r^2) = L_r^I(I) + O(r^2). \tag{4}$$

where J^I is the Jacobian of the transformation T, and $L^I(\cdot) = e(\cdot) + J^I(\cdot)r$. The operator L^I corresponds to the best linearization around the point $r = 0$ of the transformation T_r. Let R be the range of the parameter r such that $T_r(I) \approx L_r^I(I)$. If the localization condition holds for a subset of the transformation parameters contained in R, i.e.

$$\langle T_r I, t_k \rangle \approx \langle L_r^I I, t_k \rangle = 0, \quad r \notin R, \tag{5}$$

and as long as the pooling range P, in the r parameter is chosen so that $P \subseteq R$, then we are back in the group case. Thus the same reasoning used above for translation will still apply.

However this is not the case for eq. (1). The tangent space of the image's orbit is given by the Jacobian, and it clearly depends on the image itself. Since the tangent space of the image and of the template will generally be different (see Fig. 3), this prevents eq. (1) from being satisfied. More formally, for $r \in R$:

$$\langle L_r^I(I), t_k \rangle = \langle I, [L_r^I]^{-1} t_k \rangle \quad \Leftrightarrow \quad L_r^I = L_r^{t_k}.$$

That is, eq. (1) is only satisfied when the image and template "transform the same way" (see Fig. 3).

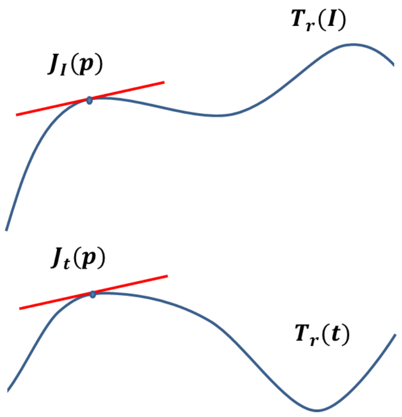

Figure 3. The Jacobians of the orbits of the image around the point p and the template must be approximately equal for eq. (1) to hold in the case of smooth transformations.

To summarize, the following three conditions are needed to have invariance for non-group transformations:

1. The transformation must be differentiable (the Jacobian must exist).
2. A localization condition of the form in eq. (5) must hold to allow a linearization of the transformation.
3. The image and templates must transform "in the same way", i.e. the tangent space of their orbits (in the localization range) must be equal. This is equivalent to $J^I \equiv J^{t_k}$.

Remark: The exposition of the theory given here is specialized for the relevant case of the general theory. In general, we allow each "element" of the signature (as defined here) to be a vector representing a distribution of one-dimensional projections of the orbit. See [2] for details.

2 Illumination invariance

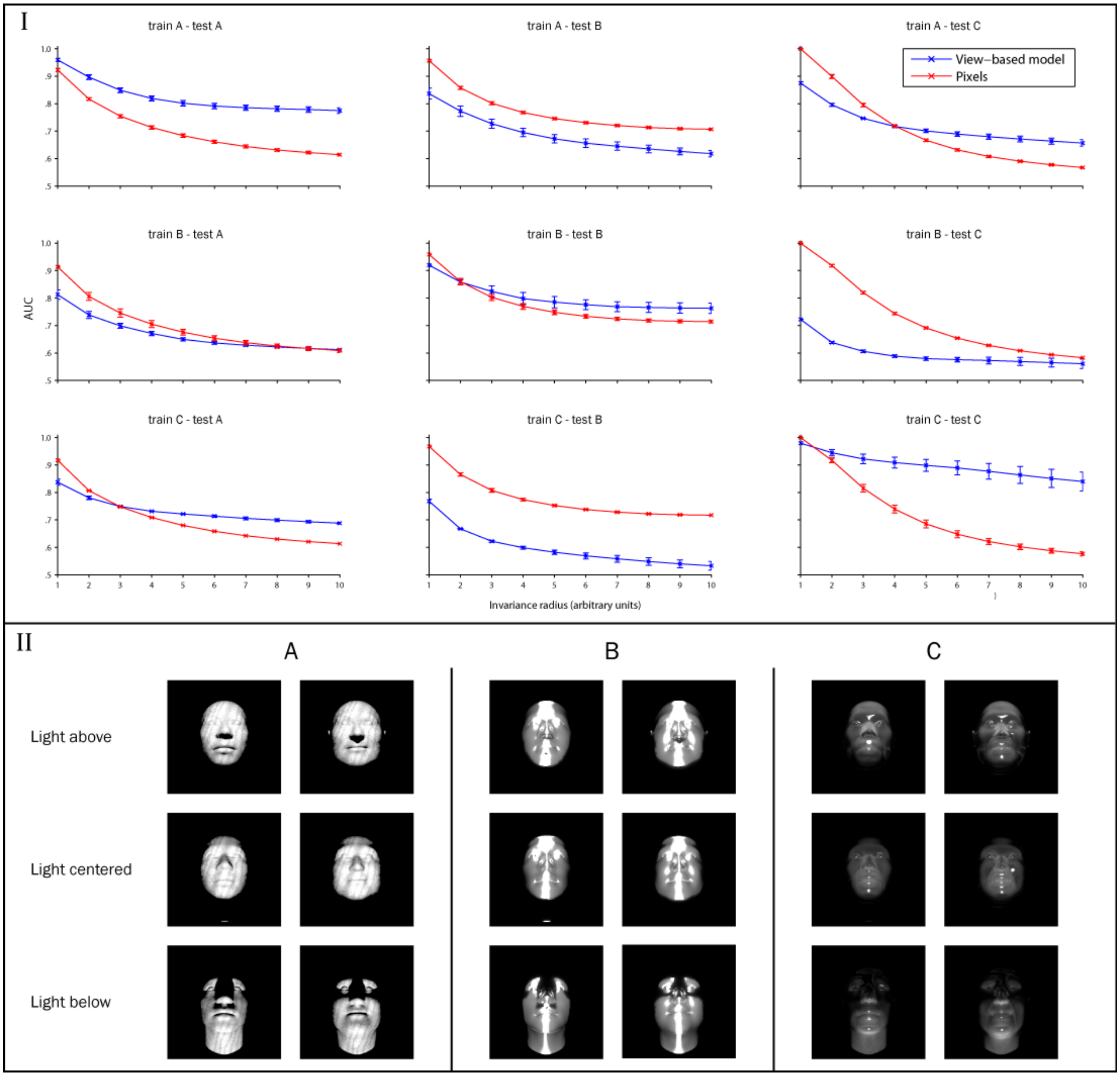

Figure 4. Class-specific transfer of illumination invariance. Bottom panel (II): Example images from the three classes. Top panel (I): The left column shows the results of a test of illumination invariance on statues of heads made from different materials (class A), the middle column shows results for class B and the right column shows the results for class C. The view-based model (blue curve) was built using images from class A in the top row, class B in the middle row, and class C in the bottom row. The abscissa of each plot shows the maximum invariance range (arbitrary units of the light source's vertical distance from its central position) over which target and distractor images were generated. The view-based model was never tested on any of the images that were used as templates. Error bars (+/- one standard deviation) were computed over 20 cross validation runs using different choices of template and test images.

Illumination is also a class-specific transformation. The appearance of an object after a change in lighting direction depends both on the object's 3D structure and on its material properties (e.g. reflectance, opacity, specularities). Figure 4 displays the results from a test of illumination-invariant recognition on three different object classes which can be thought of as statues of heads made from different materials—A: wood, B: silver, and C: glass. The results of this illumination-invariance test follow the same pattern as the 3D rotation-invariance test. In both cases the view-based model improves the pixel-based models' performance when the template and test images are from the same class (fig. 4—plots on the diagonal). Using templates of a different class than the test class actually lowered performance below the pixel-based model in some of the tests e.g. train A–test B and train B–test C (fig. 4—off diagonal plots). This simulation suggests that these object classes have high $\overline{\psi}$ with respect to illumination transformations. However, the weak performance of the view-based model on the silver objects indicates that it is not as high as the others (see the table below). This is because the small differences in 3D structure that define individual heads give rise to more extreme changes in specular highlights under the the transformation.

Object class	Transformation	$\overline{\psi}$
Glass statues	illumination	0.56320
Sliver statues	illumination	0.35530
Wood statues	illumination	0.53990

Table 1. Table of illumination transformation compatibilities

3 Pose-invariant body recognition

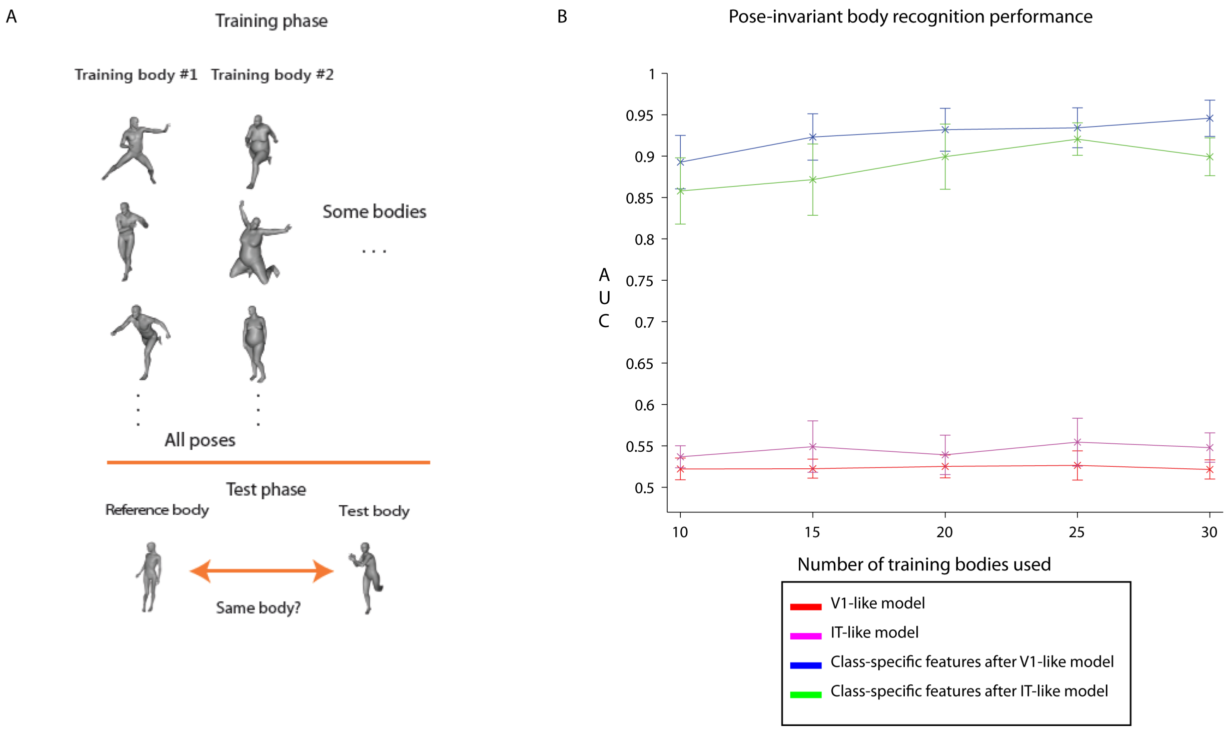

Figure 5. A. Example images for the pose-invariant body-recognition task. The images appearing in the training phase were used as templates. The test measures the model's performance on a same-different task in which a reference image is compared to a query image. 'Same' responses are marked correct when the reference and query image depict the same body (invariantly to pose-variation).
B. Model performance: area under the ROC curve (AUC) for the same-different task with 10 testing images. The X-axis indicates the number of bodies used to train the model. Performance was averaged over 10 cross-validation splits. The error bars indicate one standard deviation over splits.

Let $B = \{b_1, b_2, ..., b_n\}$ be a set of bodies and $P = \{p_1, p_2, ..., p_n\}$ be a set of poses. Let d be the dimensionality of the images. We define the rendering function $t_p : B \to \mathbb{R}^d$. In words, we say $t_p[b]$ renders an image of body b in pose p. In that case the argument b is the template and the subscript p indicates the transformation to be applied.

We obtain the signature vector $\mu : X \to \mathbb{R}^m$ by pooling the inner products of the input image with different renderings of the same template.

$$\mu(x) = \begin{pmatrix} \max(\langle I, t_1(\tau_1)\rangle, \langle I, t_2(\tau_1)\rangle, \ldots, \langle I, t_n(\tau_1)\rangle) \\ \max(\langle I, t_1(\tau_2)\rangle, \langle I, t_2(\tau_2)\rangle, \ldots, \langle I, t_n(\tau_2)\rangle) \\ \vdots \\ \max(\langle I, t_1(\tau_m)\rangle, \langle I, t_2(\tau_m)\rangle, \ldots, \langle I, t_n(\tau_m)\rangle) \end{pmatrix} \tag{6}$$

As in some HMAX implementations (e.g., Serre et al. (2007) [8]), we used a Gaussian radial basis function for the S-unit response. It has similar properties to the normalized dot product.

$$\langle I, t_i(\tau_j)\rangle = \exp\{\sigma * \sum((I - t_i(\tau_j))^2)\} \tag{7}$$

Where σ is the Gaussian's variance parameter.

The class-specific layer takes in any vector representation of an image as input. We investigated two hierarchical architectures built off of different layers of the HMAX model (C1 and C2-global) [8]—referred to in fig. 5 as the V1-like and IT-like models respectively.

For the pose-invariant body recognition task, the template images were drawn from a subset of the 44 bodies—rendered in all poses. In each of 10 cross-validation splits, the testing set contained images of 10 bodies that never appeared in the model-building phase—again, rendered in all poses (fig. 5).

The HMAX models perform almost at chance. The addition of the class-specific mechanism significantly improves performance on this difficult task. That is, models without class-specific features were unable to perform the task while class-specific features enabled good performance on this difficult invariant recognition task (fig. 5).

Downing and Peelen (2011) argued that the extrastriate body area (EBA) and fusiform body area (FBA) "jointly create a detailed but cognitively unelaborated visual representation of the appearance of the human body". These are perceptual regions—they represent body shape and posture but do not explicitly represent high-level information about "identities, actions, or emotional states" (as had been claimed by others in the literature [9]). The model of body-specific processing suggested by the simulations presented here is broadly in agreement with this view of EBA and FBA's function. It computes, from an image, a body-specific representation that could underlie many further computations e.g. action recognition, emotion recognition, etc.

4 Development of domain-specific regions

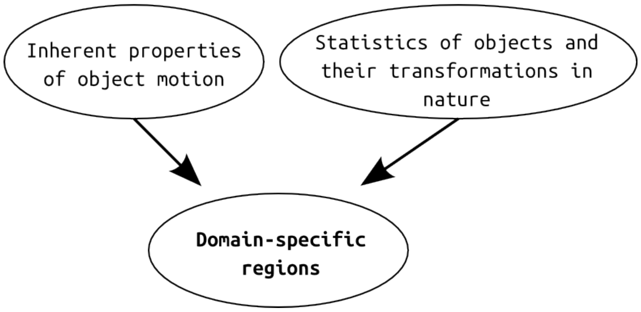

Figure 6. Two factors are conjectured to influence the development of domain-specific regions.

We consider three different arbitrary choices for the distributions of objects from five different categories: faces, bodies, vehicles, chairs, and animals (see table 2). Importantly, one set of simulations used statistics which were strongly biased against the appearance of faces as opposed to other objects.

	Name of simulation	Faces	Bodies	Animals	Chairs	Vehicles
A.	"Realistic"	76	32	16	16	16
B.	Uniform	30	30	30	30	30
C.	Biased against faces	16	32	36	36	36

Table 2. Numbers of objects used for each simulation. In the "realistic" simulation, there were proportionally more faces.

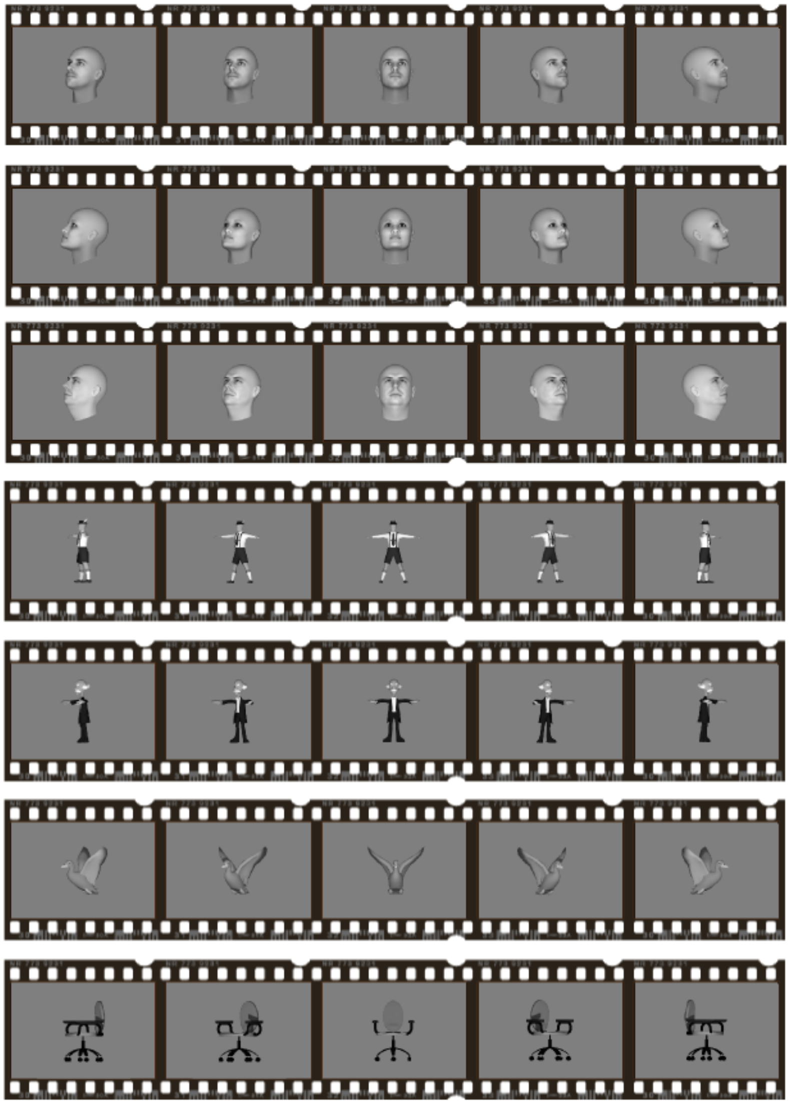

Figure 7. Example object videos (transformation sequences) used in the ψ-based clustering experiments.

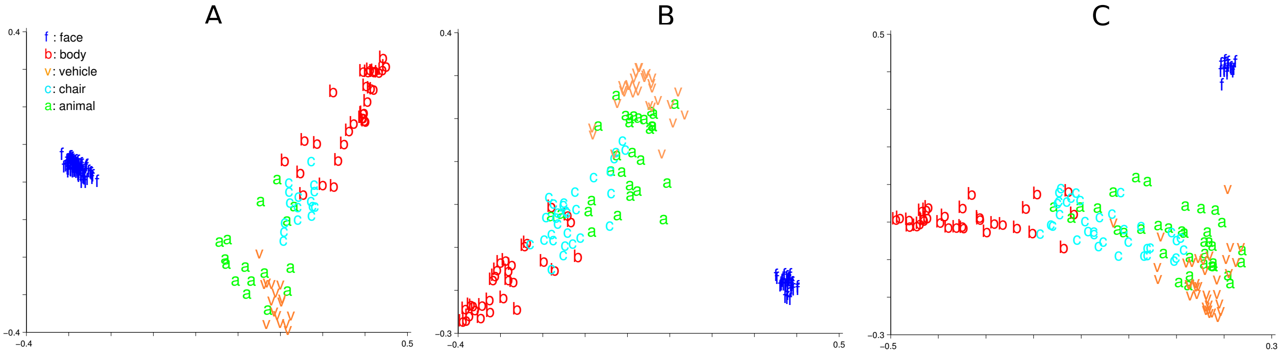

Figure 8. Multidimensional Scaling (MDS) [10] visualizations of the object sets under the $\psi(A, B)$-dissimilarity metric for the three object distributions: A. "realistic", B. uniform, and C. biased against faces (see table 2).

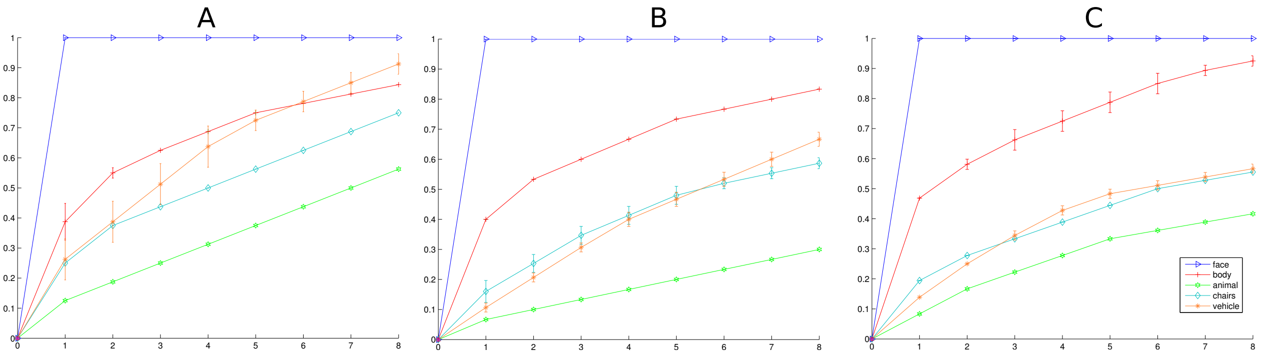

Figure 9. The percentage of objects in the first N clusters containing the dominant category object (clusters sorted by number of objects in dominant category). A, B and C are respectively, the "realistic" distribution, uniform distribution, and the biased against faces distribution (see table 2)). 100% of the faces go to the first face cluster—only a single face cluster developed in each experiment. Bodies were more "concentrated" in a small number of clusters, while the other objects were all scattered in many clusters—thus their curves rise slowly. These results were averaged over 5 repetitions of each clustering simulation using different randomly chosen objects.

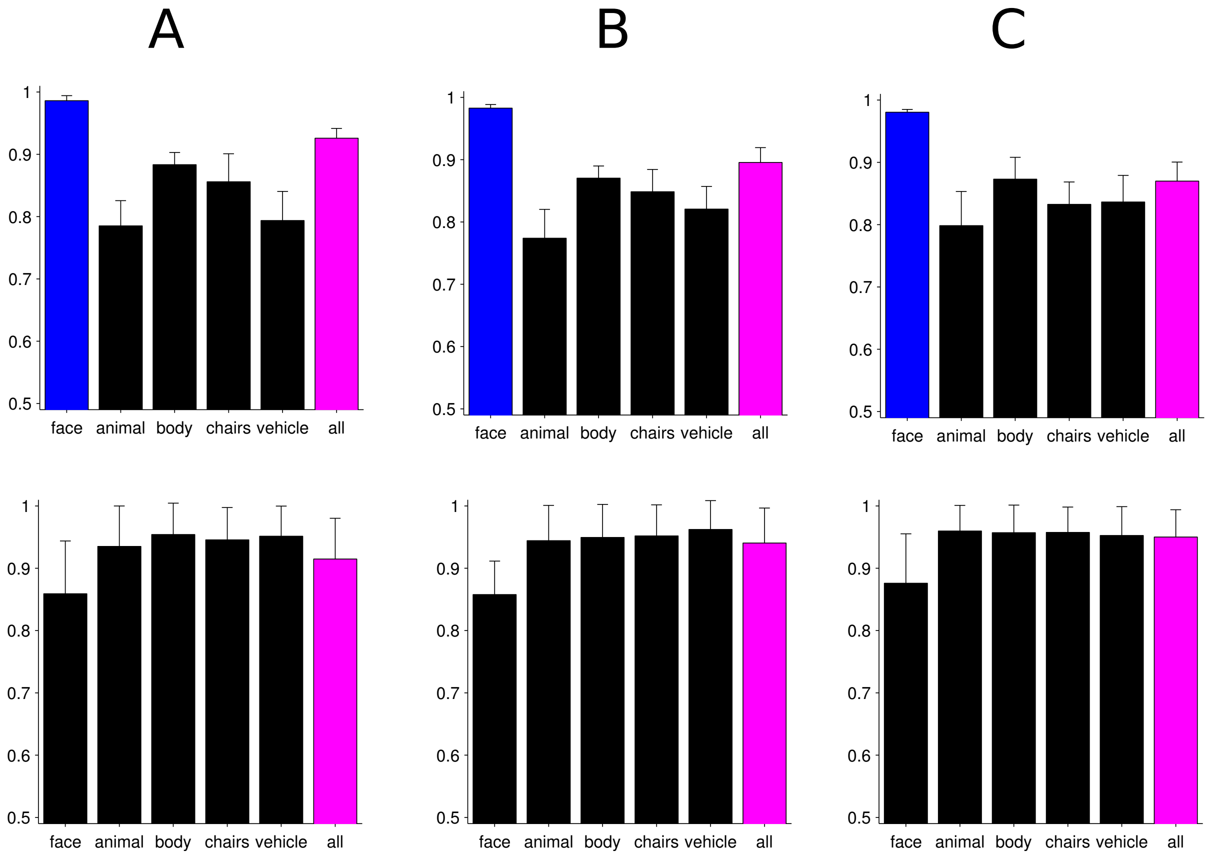

Figure 10. The classification performance on face recognition, a subordinate-level task (top row) and car vs. airplane, a basic-level categorization task (bottom row) using templates from each cluster. 5-fold cross-validation, for each fold, the result from the best-performing cluster of each category is reported. A, B and C indicate "realistic", uniform, and biased distributions respectively (see table 2). Note that performance on the face recognition task is strongest when using the face cluster while the performance on the basic-level car vs. airplane task is not stronger with the vehicle cluster (mostly cars and airplanes) than the others.

5 Supplementary methods

5.1 Stimuli

Illumination

Illumination: Within each class the texture and material properties were exactly the same for all objects. We used Blender to render images of each object with the scene's sole light source placed in different locations. The 0 position was set to be in front of the object's midpoint; the light was translated vertically. The most extreme translations brought the light source slightly above or below the object. Material data files were obtained from the Blender Open Material Repository (http://matrep.parastudios.de/). 40 heads were rendered with each material type. For each repetition of the experiment, 20 were randomly chosen to be templates and 20 to be testing objects. Each experiment was repeated 20 times with different template and testing sets.

Bodies / pose

DAZ 3D Studio was used to render each of 44 different human bodies under 32 different poses, i.e., 44*32 =1408 images in total.

5.2 Body-pose experiments

For the body-pose invariance experiments (fig. 5), the task was identical to the test for unfamiliar faces and novel object classes. The same classifier (Pearson correlation) was used for this experiment. Unlike rotation-in-depth, the body-pose transformation was not parameterized.

5.3 Clustering by transformation compatibility

Pseudocode for the clustering algorithm is given below (algorithm 1).

Let A_i be the i_{th} frame of the video of object A transforming and B_i be the i_{th} frame of the video of object B transforming. The Jacobian can be approximated by the "video" of difference images: $J_A(i) = |A_i - A_{i+1}|$ $(\forall i)$. The "instantaneous" transformation compatibility is $\psi(A,B)(i) := \langle J_A(i), J_B(i) \rangle$. Thus for a range of parameters $i \in R = [-r, r]$, the empirical transformation compatibility between A and B is

$$\psi(A,B) := \frac{1}{|R|} \sum_{i=-r}^{r} \langle J_A(i), J_B(i) \rangle \,. \quad (8)$$

The transformation compatibility $\overline{\psi}$ of a cluster C was defined as the average of the pairwise compatibilities $\psi(A,B)$ of all objects in C.

$$\overline{\psi}(C) := \text{mean}(\psi(A,B)) \;\; \text{for all pairs of objects } (A,B) \text{ from } C. \quad (9)$$

Algorithm 1 Iterative clustering algorithm interpreted as a model of ventral stream development

```
Input: All Objects: O, i_th Object: O_i where i = 1...N, Threshold: T)
Output: ClusterLabels

ClusterLabels(1) = 1
ψ̄ = computeCompatibility(ClusterLabels)
for i = 2 to N do
  ψ = computeCompatibilityWithEveryCluster(i, O, ClusterLabels)
  [MaxValue MaxIndex] = max(ψ)
  if MaxValue > T then
    ClusterLabels(i) = MaxIndex    //Assign to the cluster with the highest compatibility.
  else
    ClusterLabels(i) = max(ClusterLabels) + 1    //Create a new cluster
  end if
  ψ̄ = updateCompatibility(ψ, CurrentClusterCompatibility, ClusterLabels(i))
end for

Function   computeCompatibilityWithEveryCluster(IDX,AllObjects,ClusterLabels)
//Initialize ψ as an empty array of length #Clusters.
for i = 1 to #Clusters do
  Objects = GetObjectsFromCluster(i, AllObjects, ClusterLabels)
  for j = 1 to #Objects do
    tmpArray(j) = compatibilityFunction(AllObjects(IDX), Objects(j))
  end for
  ψ(i) = mean(tmpArray);
end for
Return ψ
EndFunction
```

References

[1] Simoncelli EP, Olshausen BA (2001) Natural image statistics and neural representation. Annual Review of Neuroscience 24: 1193–1216.

[2] Anselmi F, Leibo JZ, Mutch J, Rosasco L, Tacchetti A, et al. (2013) Unsupervised Learning of Invariant Representations in Hierarchical Architectures. arXiv:13114158v3 [csCV] .

[3] Thorpe S, Fize D, Marlot C (1996) Speed of processing in the human visual system. Nature 381: 520–522.

[4] Hung CP, Kreiman G, Poggio T, DiCarlo JJ (2005) Fast Readout of Object Identity from Macaque Inferior Temporal Cortex. Science 310: 863–866.

[5] Isik L, Meyers EM, Leibo JZ, Poggio T (2013) The dynamics of invariant object recognition in the human visual system. Journal of Neurophysiology 111: 91–102.

[6] McCulloch WS, Pitts W (1943) A logical calculus of the ideas immanent in nervous activity. The Bulletin of Mathematical Biophysics 5: 115–133.

[7] Hubel D, Wiesel T (1962) Receptive fields, binocular interaction and functional architecture in the cat's visual cortex. The Journal of Physiology 160: 106.

[8] Serre T, Oliva A, Poggio T (2007) A feedforward architecture accounts for rapid categorization. Proceedings of the National Academy of Sciences of the United States of America 104: 6424–6429.

[9] Downing P, Peelen M (2011) The role of occipitotemporal body-selective regions in person perception. Cognitive Neuroscience 2: 186–203.

[10] Torgerson WS (1958) Theory and methods of scaling. Wiley.
