# Supplementary figures and images for: The Invariance Hypothesis Implies Domain-Specific Regions in Visual Cortex
Source: PLoS Comput Biol. 2015 Oct 23;11(10):e1004390. doi: 10.1371/journal.pcbi.1004390 (PMC4619805; doi:10.1371/journal.pcbi.1004390)

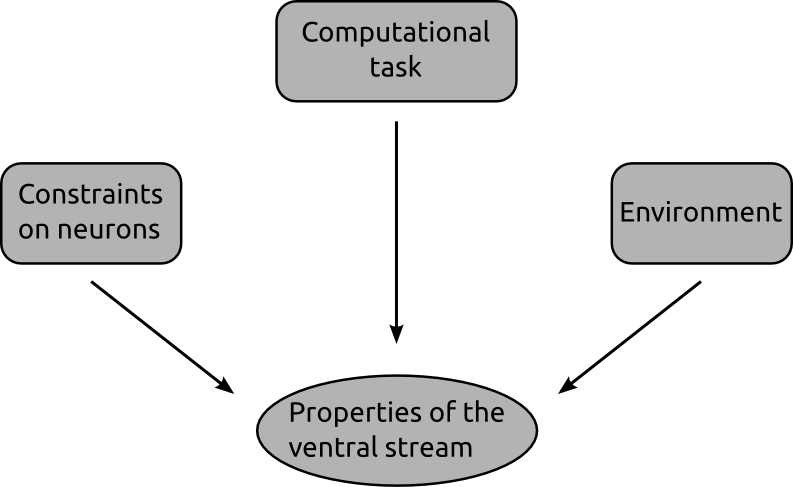

Supplement: S1 Fig — We are not the only ones to identify them in this way. For example, Simoncelli and Olshausen distinguished the same three factors [20]. The crucial difference between their efficient coding hypothesis and our invariance hypothesis is the particular computational task that we consider. In their case, the task is to provide an efficient representation of the visual world. In our case, the task is to provide an invariant signature supporting object recognition. (TIF) [file pcbi.1004390.s002.tif]

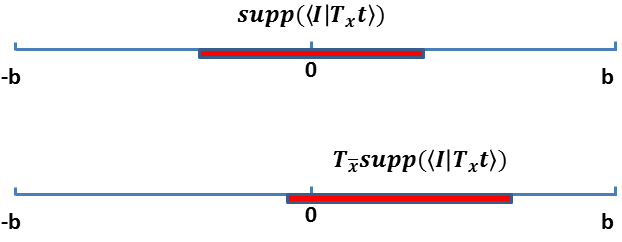

Supplement: S2 Fig — (TIF) [file pcbi.1004390.s003.tif]

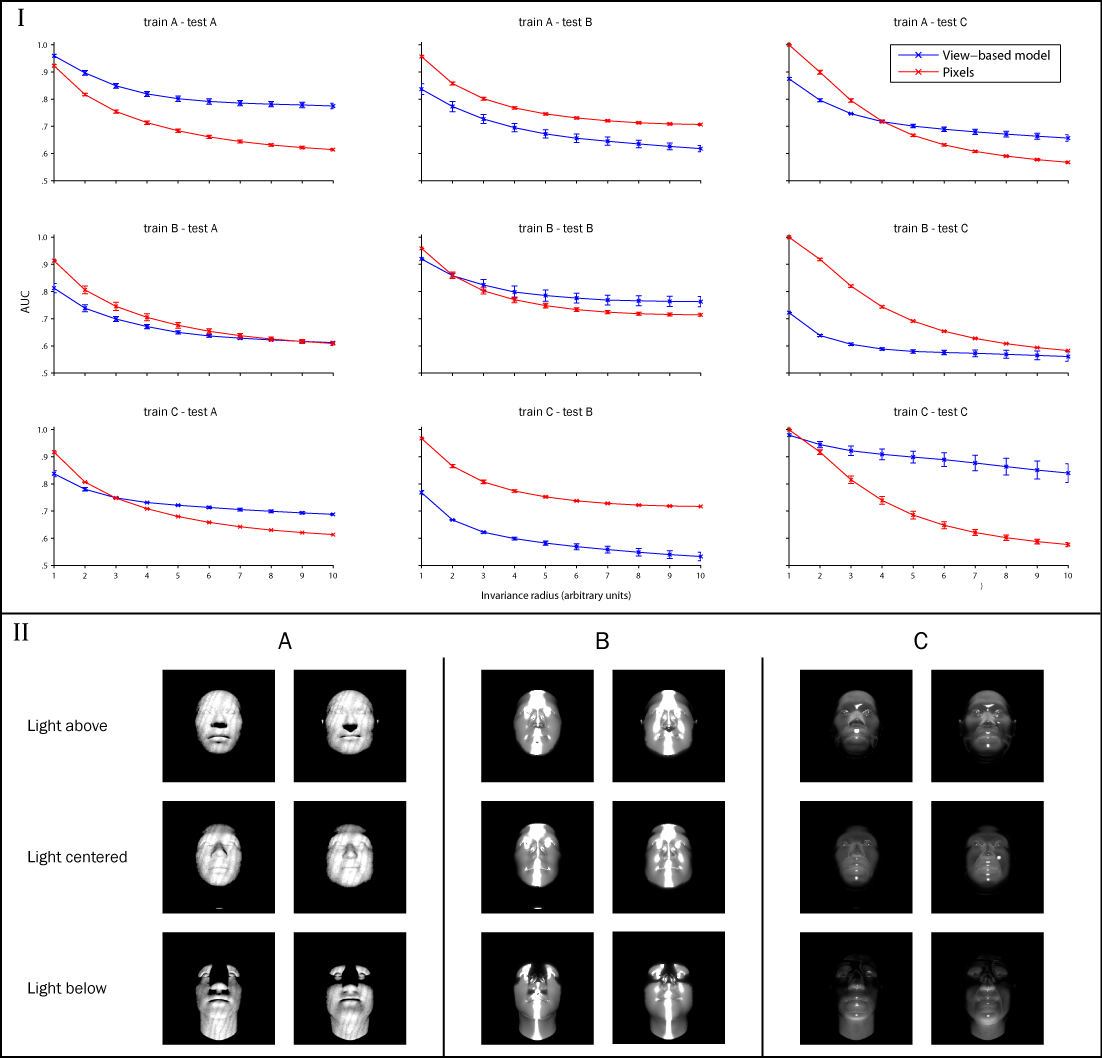

Supplement: S4 Fig — Bottom panel (II): Example images from the three classes. Top panel (I): The left column shows the results of a test of illumination invariance on statues of heads made from different materials (class A), the middle column shows results for class B and the right column shows the results for class C. The view-based model (blue curve) was built using images from class A in the top row, class B in the middle row, and class C in the bottom row. The abscissa of each plot shows the maximum invariance range (arbitrary units of the light source’s vertical distance from its central position) over which target and distractor images were generated. The view-based model was never tested on any of the images that were used as templates. Error bars (+/- one standard deviation) were computed over 20 cross validation runs using different choices of template and test images. (TIF) [file pcbi.1004390.s005.tif]

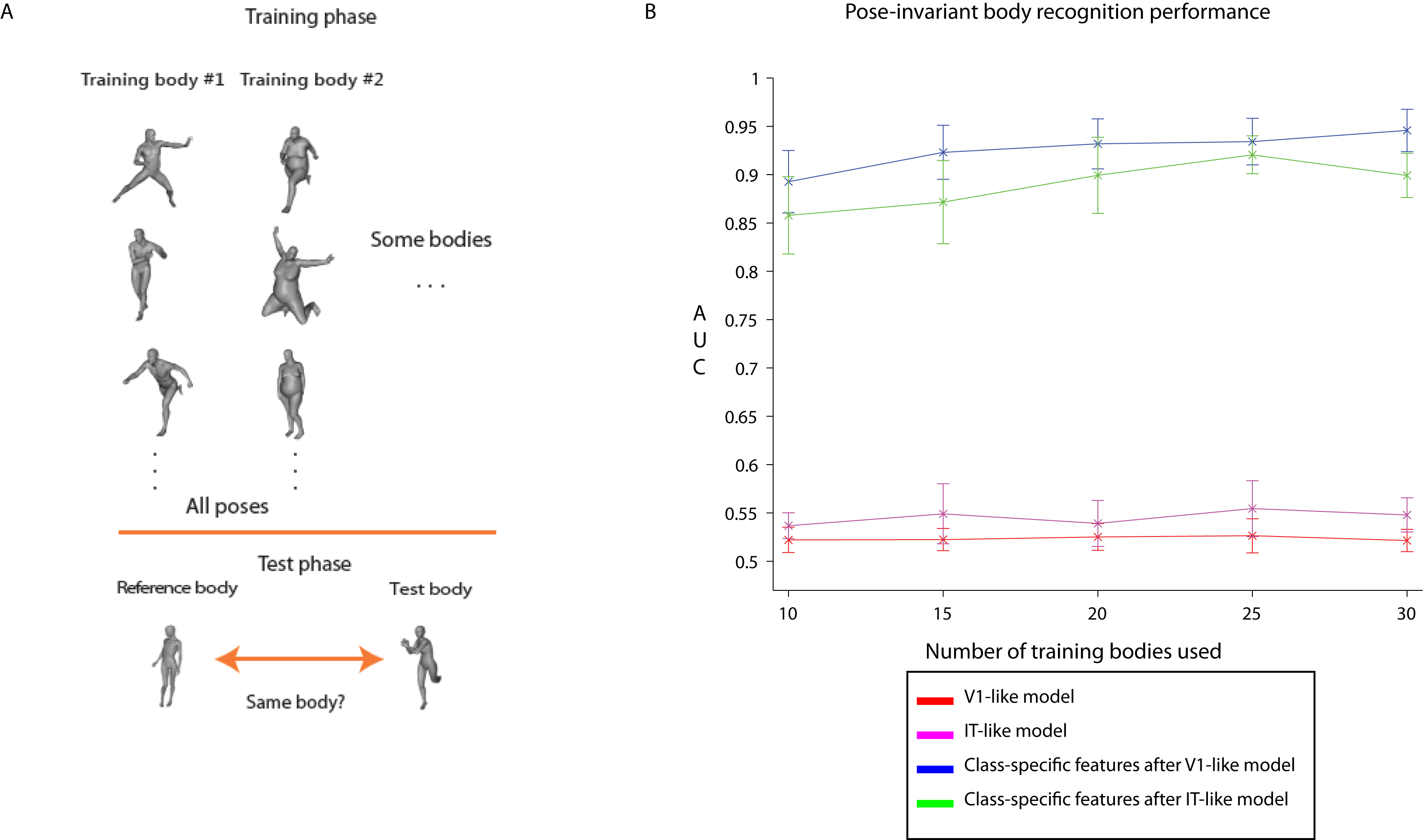

Supplement: S5 Fig — A. Example images for the pose-invariant body-recognition task. The images appearing in the training phase were used as templates. The test measures the model’s performance on a same-different task in which a reference image is compared to a query image. ‘Same’ responses are marked correct when the reference and query image depict the same body (invariantly to pose-variation). B. Model performance: area under the ROC curve (AUC) for the same-different task with 10 testing images. The X-axis indicates the number of bodies used to train the model. Performance was averaged over 10 cross-validation splits. The error bars indicate one standard deviation over splits. (TIF) [file pcbi.1004390.s006.tif]

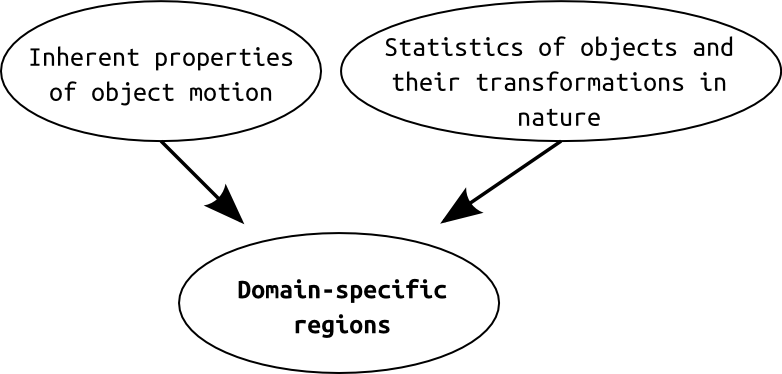

Supplement: S6 Fig — (TIF) [file pcbi.1004390.s007.tif]

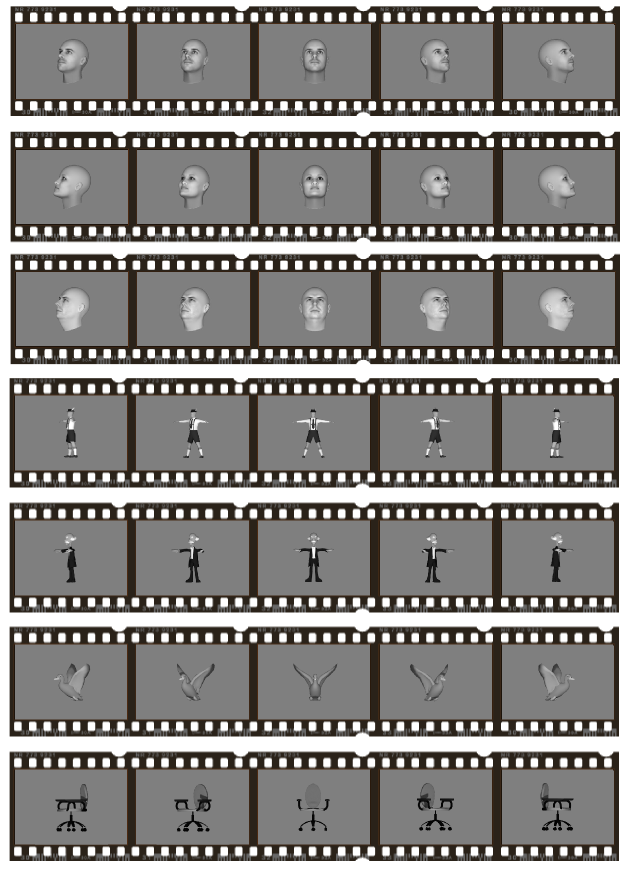

Supplement: S7 Fig — (TIF) [file pcbi.1004390.s008.tif]

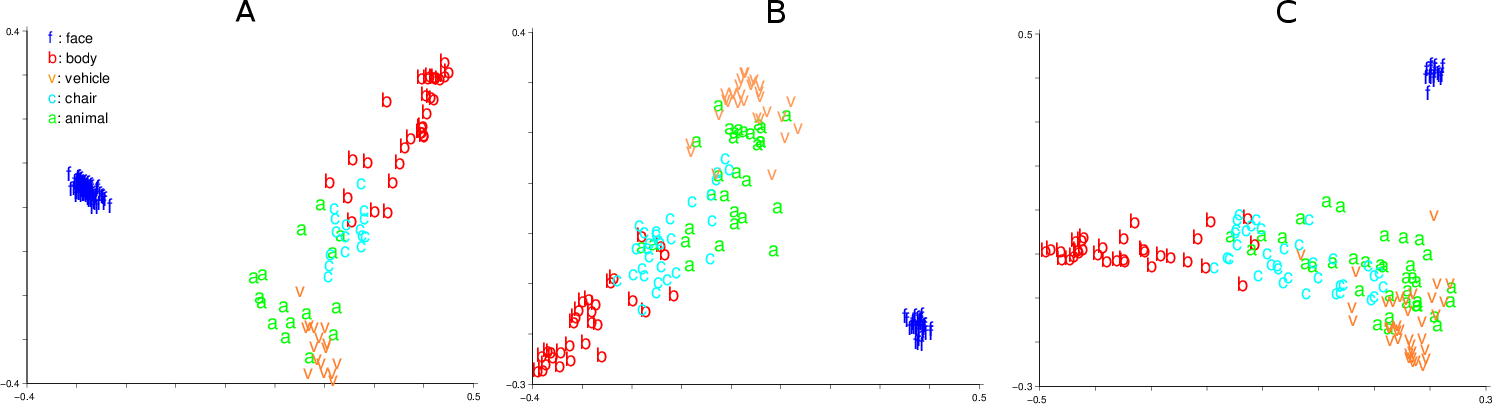

Supplement: S8 Fig — A. “realistic”, B. uniform, and C. biased against faces (see table). (TIF) [file pcbi.1004390.s009.tif]

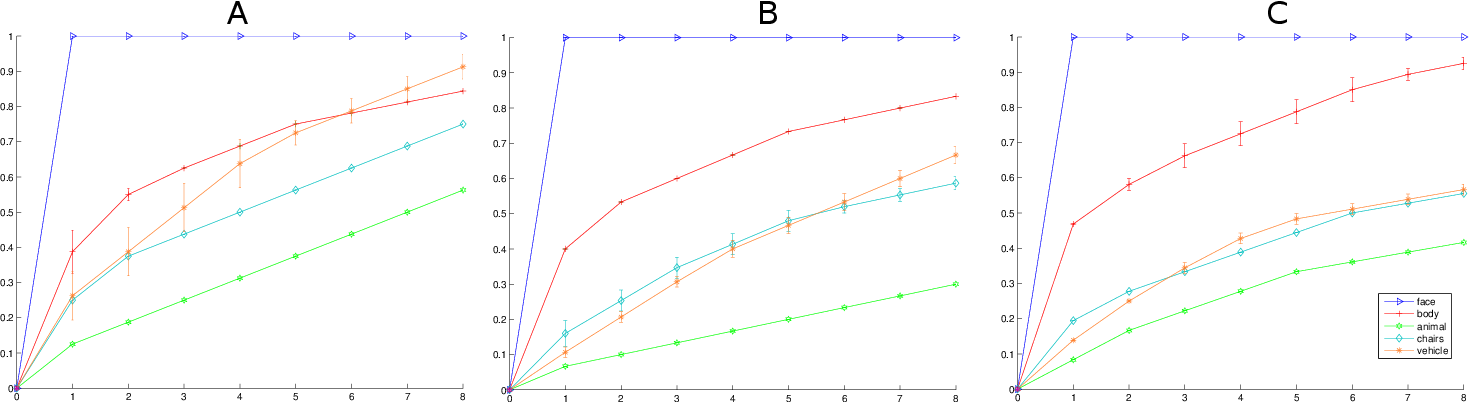

Supplement: S9 Fig — A, B and C are respectively, the “realistic” distribution, uniform distribution, and the biased against faces distribution (see table)). 100% of the faces go to the first face cluster—only a single face cluster developed in each experiment. Bodies were more “concentrated” in a small number of clusters, while the other objects were all scattered in many clusters—thus their curves rise slowly. These results were averaged over 5 repetitions of each clustering simulation using different randomly chosen objects. (TIF) [file pcbi.1004390.s010.tif]

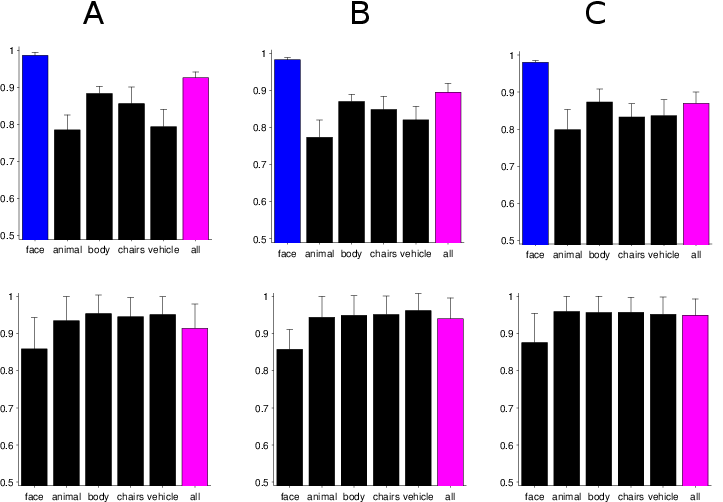

Supplement: S10 Fig — 5-fold cross-validation, for each fold, the result from the best-performing cluster of each category is reported. A, B and C indicate “realistic”, uniform, and biased distributions respectively (see table). Note that performance on the face recognition task is strongest when using the face cluster while the performance on the basic-level car vs. airplane task is not stronger with the vehicle cluster (mostly cars and airplanes) than the others. (TIF) [file pcbi.1004390.s011.tif]
